# Harmonic Lingual Frenotomy for Ankyloglossia: A Newer Novel Technique

**DOI:** 10.7759/cureus.23223

**Published:** 2022-03-16

**Authors:** Mohd Altaf Mir, Rajesh Maurya, Om Parkash, Gourav Kaushal, Nirjhar Raj Rakesh

**Affiliations:** 1 Burns & Plastic Surgery, All India Institute of Medical Sciences, Bathinda, IND; 2 Surgical Gastroenterology, All India Institute of Medical Sciences, Bathinda, IND; 3 Surgical Gastroenterology, All India Institute of Medical Sciences, Rishikesh, IND

**Keywords:** articulation, speech, frenuloplasty, frenotomy, ankyloglossia

## Abstract

The objective of this is to report a newer novel technique of harmonic scalpel frenotomy, a day care procedure under local infiltration anesthesia that could achieve quick bloodless adequate ankyloglossia release and excellent healing with subsequent improved articulation and speech. The procedure was performed, patient followed up for six months and excellent results noted. A 37-year-old male patient presented with type II ankyloglossia on Coryllos ankyloglossia grading scale and class III on Kotlow’s assessment. A quick bloodless frenotomy with adequate release of ankyloglossia was achieved using harmonic scissors. Patient experienced no discomfort. Patient was observed for two hours and discharged the same day with chlorhexidine mouth gargles and analgesics. Postoperative healing was excellent with adequate tongue protrusion and improvement in articulation and speech. We report this newer novel technique of harmonic scissors frenotomy, a day care procedure under local infiltration anesthesia, that achieved quick bloodless adequate ankyloglossia release, excellent healing and evident improvement in articulation and speech.

## Introduction

The severity of ankyloglossia may vary from little difficulty in articulation to completely unable to articulate and produce a sound and comprehensible speech. Both severity of ankyloglossia and difficulty of comprehensibility of speech are directly proportional to the type of ankyloglossia due to fine elastic which in turn depend on the thickness, shortness and extent of the frenula [1,2].

There are different surgical methods of frenotomy. However, in our case of ankyloglossia in an adult, we used a newer novel technique of harmonic scalpel frenotomy, a day care procedure under local infiltration anesthesia that could achieve quick bloodless adequate ankyloglossia release and excellent healing, which, in turn, produce an evident improvement in articulation and speech. 

## Case presentation

A 37-year-old male patient came with difficulty in protruding tongue since birth. Patient had no history of trauma in the tongue. The difficulty in articulation was evident for consonants and sounds like “z", "t", "d", "l", "dg” and difficulty to roll an “r”. He didn’t have any difficulty in feeding. On examination, the patient had stable general condition and on intraoral examination, patient had ankyloglossia with restriction in protrusion of tongue beyond the incisor teeth. The frenulum was 6 mm long, thick extending from just 3 mm proximal of the ventral side of the tongue to the floor of the mouth, hence having type II ankyloglossia on Coryllos ankyloglossia grading scale and class III on Kotlow’s assessment.

**Figure 1:**
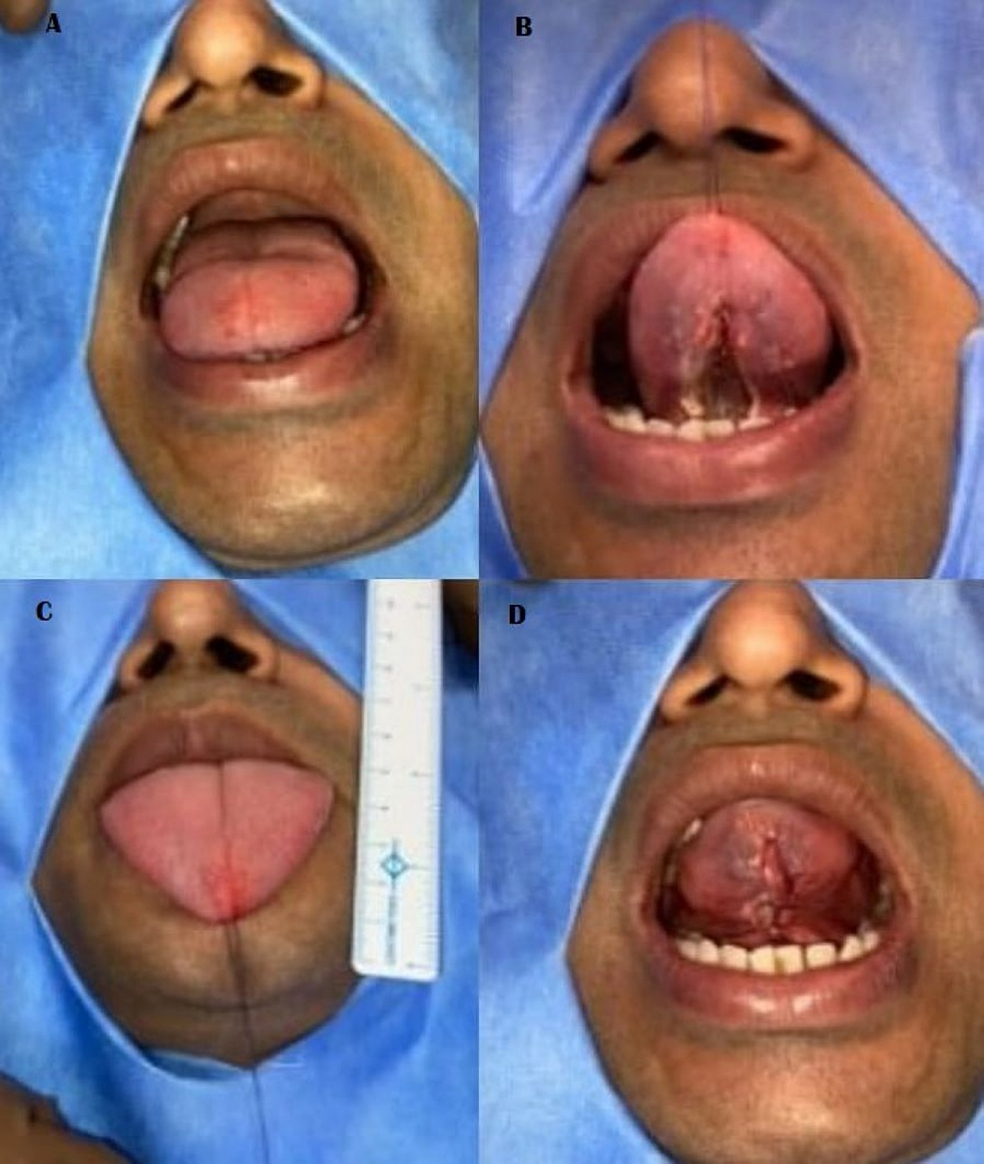
Intraoperative photographs Panel A depicts restriction in protrusion of tongue beyond the red line of lower lip. Panel B depicts frenotomy leading to diamond shaped defect and also depict the increased mobility of tongue. Panel C depicts improvement in mobility (protrusion) of tongue immediately and was checked intraoperatively both passively and actively. Panel D depicts the completion of the procedure of frenuloplasty.

A plan to release the tongue tie under local anesthesia was made and was discussed with the patient and written informed consent was obtained. Preoperative workup was done which showed the patient was fit for surgery under local anesthesia. Patient was placed in supine position on operation table with head end raised to 45 degrees. Painting and draping were done taking aseptic precautions. Infiltration was given with 1:200000 solution of lignocaine 2% with adrenaline solution in the region of frenulum and adjacent area of 1 cm width. The tip of the tongue was held using a tongue tip suture followed by assessment of tongue protrusion on pull of the suture revealing restriction in protrusion of tongue beyond the red line of lower lip (Figure 1A). The frenotomy was performed (Video 1, Figure 1B) using harmonic scissors (Ethicon) until the tongue was completely free (Figure 1C). The power used was min 3-max 5. A quick bloodless adequate ankyloglossia release was achieved. The improvement in mobility (protrusion) of tongue immediately was checked intraoperatively both passively and actively (Figure 1C). After release of thick frenulum, the diamond-shaped wound was sutured primarily with 3/0 polygalactide 910 (Vicryl) suture, completing the procedure of frenuloplasty (Figure 1D).

**Video 1:** Intraoperative video of harmonic frenotomy, also known as frenulotomy. This video depicts the release of tongue tie using harmonic energy.

**Figure 2:**
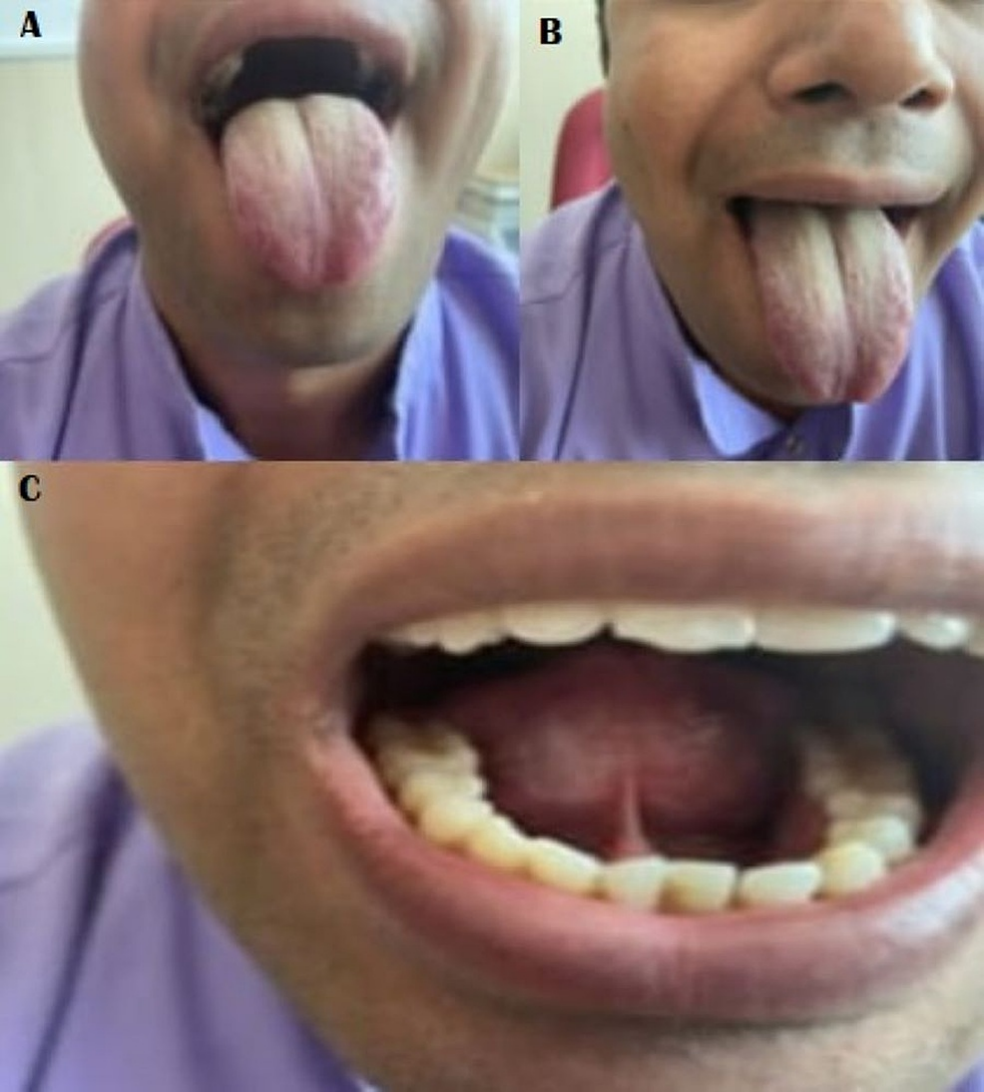
Postoperative assessment photographs. Panel A depicts improved mobility of tongue postoperatively before start of tongue exercise protocol. Panel B depicts much more improvement in tongue mobility after the completion of tongue exercise protocol. Panel C depicts the normal looking neo-frenulum after complete healing and tongue exercises.

The procedure was completed within 10 minutes after infiltration anesthesia. Patient experienced no discomfort during the procedure, was observed for two hours and discharged the same day with chlorhexidine mouth gargles and analgesics. There were no intraoperative or postoperative complications. The patient was called on postoperative days seven and 14 for follow-up. The excellent healing and improved tongue movements (Figure 2A) were observed and patient was advised to perform tongue release post-operative exercises to reorient the immature collagen into mature collagen, prevent fibrosis by stretching and hence the excellent healing and evident improvement in articulation and speech was achieved. The last follow-up examination was performed six months later and patient had excellent healing, free tongue with adequate protrusion (Figure 2B), speech articulation and normal looking neo-frenulum (Figure 2C).

## Discussion

Tongue tie (ankyloglossia) is a quite common condition affecting more than 4-11% of newborn infants [2]. The lingual frenulum is a mucous membrane fold extending from the midline of the ventral surface of the tongue to the floor of the mouth. It helps in stabilizing the base of the tongue. Normally it seldom interferes in the movement of the tongue [3], however, in cases of ankyloglossia the frenulum has an attachment more anteriorly near the tip of the tongue, which is thick, short and tight. This causes restriction in the movement of the tongue leading to feeding difficulties and difficulties in speech articulation [4]. In addition to these in older children and adults it has been reported to cause abnormal dentition and poor oral hygiene [5]. However, our patient only had difficulty in articulation. He had difficulty in articulation and producing speech sounds like “z", "t", "d", "l", "dg” and difficulty to roll an “r”. His dentition was normal, and he had impeccable oral hygiene.

Ankyloglossia is classified according to severity into 4 grades by Coryllo [6]. In type I frenulum is thin and elastic and anchors the tip of the tongue to the ridge behind lower teeth. Type II has fine and elastic frenulum and the tongue is anchored 2 - 4 millimeters from the tip to the floor of the mouth close to the ridge behind the lower teeth. In type III the frenulum is thick and stiffened, and anchors the tongue from the middle of the underside to the floor of the mouth. In the case of type IV the frenulum is posterior or not visible, but when touching the area with the fingertips, the examiner can feel tight fibers anchoring the tongue, with or without a thickened, shiny surface on the floor of the mouth.

The ankyloglossia is also classified into four classes based on Kotlow’s assessment. Class I (mild ankyloglossia), class II (moderate ankyloglossia), class III (severe ankyloglossia) and class IV (complete ankyloglossia) are characterized with frenulum length of 12 to 16 mm, 8 to 11 mm and 3 to 7 mm and less than 3 mm respectively [7].

Frenotomy is the most common procedure for correction of ankyloglossia as it is simple, quick and can be done as an outpatient procedure [8]. Various techniques of frenotomy have been defined in literature ranging from cold dissection, electrocautery to lasers. All these procedures are simple and bloodless. But the limitation of these procedures is the chances of recurrence due to which the patients might require revision surgery in the form of frenotomy and frenuloplasty [6,8]. In our case the harmonic scissor has been used to perform the bloodless frenotomy to release the ankyloglossia in an adult with higher grade of ankyloglossia under local infiltration anesthesia. Harmonic scissor uses ultrasonic energy which gets converted into mechanical energy which cut and coagulate the vessels up to 2 mm in size simultaneously without lateral dissipation of thermal energy and hence prevent the collateral damage with subsequent better healing and little chances of recurrence. There is also no need to apply hemostatic ligature which otherwise would block the salivary gland ducts. Our patient was followed up for six months and has had no signs of recurrence.

In literature to date there is no reported use of harmonic scissors for frenotomy for ankyloglossia release in adults with any grade of ankyloglossia. However, harmonic scalpel, laser and cold dissection have been used to release the tongue tie in both adults and pediatric cases of ankyloglossia. Literature search done in PubMed, Scopus and Web of Science does not reveal use of harmonic scissors to release ankyloglossia in adults and pediatric cases. However, on searching the literature on Google Scholar we could find one pediatric case of an 11-month-old boy with lower grade ankyloglossia released with harmonic scissors under general anesthesia [9]. What is known and what is new is summarized in Table 1.

**Table 1:** What is known and what is not known. The table depicts the facts which are known so far and the gaps in the literature regarding frenotomy and frenuloplasty. It also shows the advantages of harmonic frenotomy.

What is Known
Various techniques of frenotomy have been defined in literature ranging from cold dissection, electrocautery to lasers. The harmonic scissor has been used in a single 11-month-old pediatric case of low grade ankyloglossia under general anesthesia [9].
What is New
In our case the harmonic scissor has been used first time in an adult case of higher grade ankyloglossia to perform the bloodless frenotomy to release the ankyloglossia under local infiltration anesthesia without recurrence. There is also no need to apply hemostatic ligature which otherwise would block the lingual salivary gland ducts.

## Conclusions

In conclusion we can say that frenotomy can be safely and effectively performed using harmonic scissors. The operating time is reduced, is performed under local infiltration anesthesia in adults and short general anesthesia in pediatric age group with no blood loss. There is also no need to apply hemostatic ligature which otherwise would block the salivary gland ducts. There is minimal postoperative pain, excellent healing and no recurrence.
